# The History of a Case of Traumatic Tetanus, Successfully Treated

**Published:** 1820-03

**Authors:** J. C. Montani

**Affiliations:** Villa-Sariola.


FOR THE LONDON MEDICAL AND PHYSICAL JOURNAL.

### *The History of a Case of Traumatic Tetanus, successfully Treated.* By Dr. J. C. Montani, of Villa-Sariola.

ANTHONY BRUNETTI, fifty-eight years of age, of a bilious temperament, living at Torricella, in the province of Mantua, had enjoyed vigorous health throughout the course of his laborious life, excepting some accessions of intermittent fever.

On the 9th of March, whilst pruning his vines, he wounded himself on the back of the left hand, between the thumb and index-finger, and near the extensor tendon of the latter. The wound was about an inch in length and two lines in depth: it was not attended with severe pain, but in the first instance with very abundant hemorrhage. This ceased on the edges of the wound being brought in contact with the application of pressure, but burst out anew several times during the first two days. At length, finding he had lost a great quantity of blood, (which he supposed to be about three pounds and a half,) Brunetti got the assistance of a surgeon, who by proper measures suppressed the hemorrhage. The wound cicatrized with the greatest rapidity, without the occurrence of regular suppuration. The patient then resumed his rustic labours; when, on the 30th, (the 21st day from the wound,) he perceived that the motions of his left arm were performed with difficulty, and that the little finger of this arm was strongly contracted and bent into the palm. On the ensuing day, the other fingers were in the same state, the thumb alone remaining free. These circumstances, however, which might have seriously alarmed another person, did not prevent this hardy peasant from continuing his labour until the 3d of April. On the night which preceded this day, after having had some hours of tranquil sleep, he experienced, on waking, a general difficulty of voluntary motion, with almost insupportable pain, at first confined to the muscles of the neck and shoulder already contracted, but which extended in a few hours to those of the back and loins, and rendered the movements of the head, the lower jaw, and the trunk, extremely difficult. He was obliged to lie in bed; and, in the course of the day above mentioned, almost all the muscles were in such a state of contraction, that all voluntary motion was abolished; and he suffered transient, but very severe, convulsions all over the body, and particularly at the lower part of the sternum. It was at this time that I was required to visit him. I found him lying across the bed, his head violently drawn backwards, and the lower jaw so much closed, that it with difficulty permitted the introduction of liquids into the mouth. Deglutition was effected with great trouble, and the voice was almost unintelligible. The eye was fixed, the pupil contracted, and the traits of the face, which was covered with an abundant sweat, were altered in an extraordinary manner. The convulsive contractions of the muscles rapidly succeeded each other, especially when the patient attempted (as well as his powers would permit) to change his position. The abdomen was hard as stone; the alvine dejections were suppressed; the urine was evacuated with difficulty; the pulse was rather frequent, and very much contracted.

After having purged the patient with manna, I prescribed opium, in the dose of two grains every two hours; a warm bath to be used twice a-day, and purgative glysters to be injected.

I saw him again on the following day. He had taken twelve of the opium-pills, and he had been carefully bathed, but there was no amelioration of his state. The convulsive contractions of the muscles were, indeed, more forcible and frequent. The bath, however, gave him entire relief temporarily; for, as soon as he was plunged into it, he experienced a sensation of ease and general calmness. Although I had reason not to be content with the use of the opium, yet, as no derangement of the intellects had been produced, and as I wished to know whether this medicine was beneficial or hurtful in this case, I thought it proper to continue the administration of it: the same quantity was therefore ordered, combined with a drachm of camphor. The bath was directed to be resorted to three times a-day, if possible.

On the eighth day, matters had become still worse. The convulsions were very frequent, the face was red, the eye glistening, the pupil very contracted, the excretions almost entirely suppressed; the urine flowed in drops, and with great suffering to the patient. This deplorable state made me perceive my error; and I immediately determined to change my mode of treatment, being persuaded that the disease depended on a sthenic diathesis. I had recourse to contra-stimulants, amongst which I gave the preference to hyoscyamus; doses of twenty grains of the extract of which, divided into pills of two grains each, were ordered to be given every third hour. Had the season permitted it, I would willingly have tried the cold bath, after the advice of Hippocrates; but I confined myself to the use of a tepid one. Convinced of the necessity of obtaining alvine evacuations, I prescribed strongly-purgative glysters, to be repeated every five or six hours. The left hand and arm were enveloped in an emollient cataplasm; and a decoction of dog's-grass (*gramen*, Tourn.), with a little nitrate of potash, ordered for common drink.

The ensuing day (the ninth) I found him a little better. The fibres had certainly experienced the agency of the contra-stimulants, because the convulsive contractions of the muscles were already less frequent. The urine flowed with facility, and several evacuations from the bowels had been produced. As he had great difficulty in swallowing the pills, I ordered the extract of hyoscyamus, the dose of which was extended to half a drachm, to be dissolved in infusion of valerian, and the other measures of the preceding day to be continued.

On the tenth day, the alleviation of the disease was still more manifest. The patient was without fever; he opened his mouth with more facility; but the constipation of the bowels had reappeared. The use of the bath and the mixture was continued, without any augmentation of the quantity of the hyosciamus.

I could not see the patient again until the thirteenth day, and then I found him in a state which I believed to be desperate. The contraction of the muscles of the whole of the extremities was at its highest degree, and the convulsions succeeded each other almost without intervals. The mouth would admit only a few drops of liquid, which were soon rejected. Some words pronounced with difficulty announced derangement of the intellects. I then determined to employ the most powerfully-debilitant means. I first ordered a bleeding of about ten ounces to be practised under my own observation, and I then quitted the patient. I saw him again in the evening, and was much surprised to find him better after the use of an uncertain measure, the effects of which I had much feared. The blood was covered with a thick and tenacious buffy coat. Encouraged by this fortunate result, I had the bleeding repeated; and, as it had produced a slight relaxation of the elevator muscles of the lower jaw, I prescribed, acidulated tartrate of potash, two drachms; jalap, one drachm and a half; scammony, two grains; nitrate of potash, one drachm and a half: to be taken in six doses, after intervals of three hours. The baths and purgative glysters to be continued.

The fourteenth day, every thing had a better aspect. The powders had procured abundant alvine evacuations. The patient, having recovered mental sanity, spoke better, and could enjoy a few minutes' sleep: he made a few steps about the room, too; but he was obliged to be sustained to get to the bath. The pulse remained hard and vibrating. Eight ounces more blood were drawn; and the purgative powders were ordered to be repeated, only after longer intervals.

The fifteenth and sixteenth, the patient was still bled, and the use of the purgative powders persevered in. On the seventeenth and eighteenth, he however remained stationary. Why, I demanded of myself, have the muscular contractions diminished in duration and intensity, and yet not entirely ceded? and why are they ready to appear with their former violence whenever the wounded hand is touched, the muscles of which have never ceased to be contracted? It is, without doubt, because there exists a cause which perpetuates the disease. On this argument, I felt disposed to engage a surgeon to open again the part which had been wounded, but long since cicatrized, in order that any nervous filament, or any other very sensible part which might have been injured, might be entirely divided, and to excite an abundant suppuration; but I determined to try first the external use of mercury. I ordered, consequently, that frictions with a drachm of mercurial ointment should be made, first on the back of the affected hand, then on the limbs and on the sides of the vertebral column. The purgative powders and tepid baths were again prescribed.

On the nineteenth, there was sensible amelioration. The frictions on the back of the hand and the interior of the fore-arm, had produced relaxation of the muscles of this limb and of the whole body. The convulsions let the patient enjoy more than fifteen minutes of respite at a time, during which he could sleep. The lower jaw, less drawn upwards, permitted the introduction of food and medicines. Abundant alvine evacuations had taken place several times during the day, and much relieved the patient, whose physiognomy had resumed nearly its natural state. The pulse was regular and less tense, and the abdomen less painful. The same measures were continued.

The state of the patient during the twentieth, twenty-first, and twenty-second, days, was still more satisfactory: he could now perform motions with the trunk without pain. The muscles of the wounded hand and the arm were now almost completely relaxed. The appetite for food, which had never left the patient, even at the time of greatest danger, became excessive. As no symptom indicated the appearance of salivation, the mercurial frictions were repeated twice a-day, and a drachm and a half of the ointment was the quantity used at each time. The bath was employed only once daily; and, as the patient still complained of some difficulty in voiding the urine, I prescribed him four doses of a cooling powder.

On the twenty-fifth day, the convulsions had entirely ceased, and the patient had only some remains of trismus. Salivation had taken place. The frictions were suspended. The purgative powders and the baths continued.

The trismus entirely ceased on the twenty-ninth day, and the salivation had begun to diminish; but, as this had affected the gums and the mucous membranes of the throat, I ordered a gargle. The purgative powders also were directed to be continued. The patient left his bed for the first time; and at length, on the 3d of May, he found himself perfectly well.

